# Functional sensory function recovery of random-pattern abdominal skin flap in the repair of fingertip skin defects

**DOI:** 10.3892/etm.2012.877

**Published:** 2012-12-24

**Authors:** YA-DONG YU, YING-ZE ZHANG, WEI-DONG BI, TAO WU

**Affiliations:** Department of Hand Surgery, The Third Hospital of Hebei Medical University, Shijiazhuang, Hebei 050051, P.R. China

**Keywords:** free flap, finger tip, cutaneous deficiency, sensory function

## Abstract

The fingertip skin defect is a common hand injury often accompanied by tendon or bone exposure, and is normally treated with flaps. The aim of this study was to evaluate the functional sensory recovery of random-pattern abdominal skin flap in the repair of fingertip cutaneous deficiency. A total of 23 patients, aged between 18 and 50 years (mean age, 31 years) with fingertip cutaneous deficiency (30 digits) were treated with random-pattern abdominal skin flaps. The post-debridement defect area measured from 0.7×1.2 to 2.5×3 cm. The flap pedicle was divided three weeks after surgery, which marked the onset of the second stage. A second surgery was performed on 2 patients after 3 months and on another set of 2 patients after 6 months to create a thinner flap. Tissue was dissected during surgery for a histological examination. All the flaps survived and the post-operative follow-up ranged from 2 weeks to 6 months. Patients were satisfied with the appearance of their fingers and the flaps. All flaps demonstrated satisfactory flexibility and texture and sensory recovery was achieved. Only 4 patients were subjected to a second surgery to make the flap thinner. The flaps for the 3-month tissue section had several low-density, free nerve endings, whereas those of the 6-month section had more intensive free nerve endings, nerve tracts, tactile cells and lamellar corpuscles. Random-pattern abdominal skin flap therefore repairs fingertip skin defects achieving sensory recovery.

## Introduction

Currently, the majority of occupations in China largely involve the use of hands. The incidence of hand injury increases annually due to low-level consciousness for self-protection. The fingertip skin defect is one of the most common hand injuries that is often accompanied by tendon or bone exposure and is usually treated with flaps. Common flap types include the random-pattern abdominal flap, fascial pedicle dorsal flap of the finger, advanced skin flap and cross-finger flap. Previous studies focused on skin flap survival until the interest shifted to sensory recovery in flaps. This change occurred with the increase in patient demand for a high quality of life, as well as rapid developments in microsurgery in recent years. According to Bunnell, the founder of hand surgery, individuals with a loss of sensation in their hands have difficulty lifting small objects and holding them. At an annual meeting of American hand surgeons, Moberg compared a hand without feeling to a hand without a purpose (1). The loss of sensory function in the hand affects the movement and perceptive functions of the hand, thus leading to poor judgment and a tendency for injury. A total of 23 patients with fingertip cutaneous deficiency (30 digits) were treated with random-pattern abdominal skin flap from September 2010 to February 2011. Functional sensory recovery of the flaps in these patients was observed, and four cases were subjected to histological examination.

## Materials and methods

### General data

A total of 23 patients, 12 males and 11 females aged between 18 and 50 years (mean age, 31 years), participated in this study. All patients presented with a traumatic fingertip skin defect accompanied by tendon or bone exposure, as well as complete, partially incomplete or missing nail. This study was conducted in accordance with the declaration of Helsinki and with approval from the Ethics Committee of the Third Hospital of Hebei Medical University. Written informed consent was obtained from each participant prior to emergency surgery being performed. Among the causes of injury were an incised wound in 8 patients, a dog bite in 1 patient and mangling in 14 patients. The injured fingers included 5 thumbs, 8 index fingers, 12 middle fingers, 2 ring fingers and 3 small fingers. The defect area contained finger pulp defects in 20 fingers, dorsal digital defects in 3 fingers, finger lateral defects in 2 fingers, distal transection cut in 3 fingers and distal degloving injury in 2 fingers. The dimensions of the defect area ranged from 0.7×1.2 to 2.5×3 cm post-debridement, whereas those of the skin flap incision ranged from 1×1.5 cm to 2.8×3.3 cm.

### Surgical procedure

Under digital nerve block, the patients were subjected to radical debridement by which foreign bodies and necrotic tissues were removed without affecting the vitality of the organization. Subsequently, the two arteria digitalis were ligated. After the bleeding was stanched, the random-pattern abdominal flap was constructed based on the shapes and sizes of the wounds. Specific interventions were performed to relieve the affected limb. The skin flap area was 15–20% larger than the actual wound area to avoid excessive tension. The length-to-width ratio of the flap was maintained at 2:1 or lower to ensure flap revascularization and to allow accumulation of subdermal plexus as well as a certain amount of fat on the flap. A normal flap color was observed prior to skin flap and skin edge suture and the donor sites were directly closed. The patient was positioned in a manner that allowed the torsion of the skin flap pedicle to be prevented. The flap pedicle was divided three weeks post-debridement during the second stage.

### Follow-up content and detection methods

#### General data

Each flap was examined for tactile, pain and temperature sensation, as well as for two-point discrimination in the second week and the first, third and sixth post-operative months.

#### Detection of tactile, pain and temperature sensation

The patients were instructed to close their eyes. Detection of tactile sensation was performed by touching the skin with a small bundle of cotton. Pain sensation detection was performed by lightly puncturing the skin with a 2 ml syringe needle and temperature sensation by touching the skin with two test tubes, one of which was filled with cold water (0–10°C) and the other with hot water (50–60°C).

#### Detection of two-point discrimination

The patients were initially instructed to keep their hands still and close their eyes. The detector (blunt compasses) stimulated the flap from the proximal to the distal end, followed by a gradual decrease in the distance of the detector feet. The patients were instructed to immediately report any sensation felt (one or two points). The procedure was repeated with decreased distance until the patients were unable to distinguish the two separate stimuli. The shortest distance was recorded.

#### Sensory function evaluation

The sensory function was evaluated according to the British Medical Research Council classification: S4, normal sensitivity; S3+, recovery of useful discriminatory sensitivity; S3, recovery of complete tactile sensitivity without dysesthesia and rough useful discrimination; S2, recovery of superficial painful and incomplete tactile sensitivity with hyperesthesia and/or dysesthesia; S1, recovery of deep painful sensitivity and S0, no sensitivity.

#### Test method

Several flap tissues were collected during the flap thinning of the four fingers. The tissues were fixed with 10% formalin and then studied with hematoxylin and eosin, as well as immunohistochemical stains under a light microscope.

#### Statistical analysis

The results of two-point discrimination in the third and sixth post-operative months were presented as mean ± standard deviation. Statistical analysis was conducted using the SPSS 11.0 software (SPSS Inc., Chicago, IL, USA) and the t-test was adopted for comparison of the results between the two groups. P<0.05 was considered to indicate a statistically significant difference.

## Results

### 

#### Clinical observation

A total of 30 flaps survived. The postoperative follow-up period ranged from 2 weeks to 6 months. The appearance of the finger and the flap, as well as the color, flexibility, texture and sensory recovery were satisfactory (Table I). Only 4 finger flaps were subjected to thinning surgery, due to a bloated flap. Sensory recovery was rated S3+ and no significant donor area complications occurred.

#### Morphological observations

**Figure 1.**
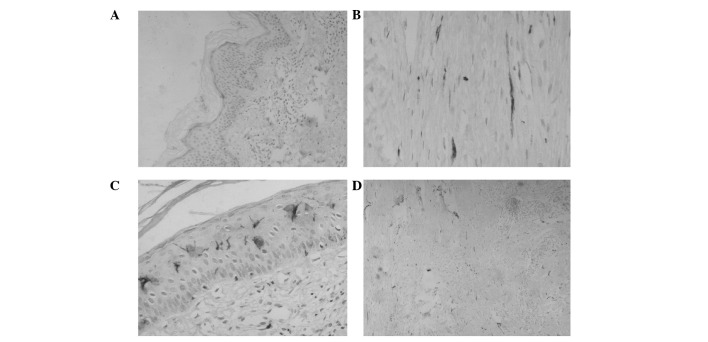
(A) Free nerve endings in the epidermal layer were scarce in the third month after surgery (magnification, ×40). (B) Nerve fibers were observed in the plexiform layer of the dermis with a lower density in the third month after surgery (magnification, ×100). (C) An abundance of free nerve endings were observed in the epidermal layer in the sixth month after surgery (magnification, ×100). (D) An abundance of nerve fibers with a greater density in the layer of dermis in the sixth month after surgery was observed (magnification, ×40). S-100 protein staining.

**Figure 2.**
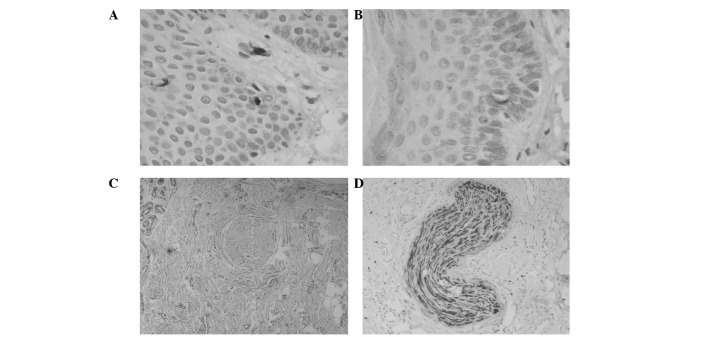
(A) A tactile corpuscle was observed in the papillary layer of the dermis in the sixth month after surgery (S-100 protein staining, magnification, ×200). (B) A Merkel cell was observed in the basal layer of the epidermis in the sixth month after surgery (S-100 protein staining, magnification, ×200). (C) Morphological integrity of the Pacinian corpuscle was observed in the deep layer of the dermis in the sixth month after surgery (HE staining, magnification, ×200). (D) Morphological integrity of the Pacinian corpuscle was observed in the deep layer of the dermis in the sixth month after surgery (S-100 protein staining, magnification, ×200). HE, hematoxylin and eosin.

In the third post-operative month, the amount of free nerve endings in the epidermal layer of the flap slice was minimal (Fig. 1A) and the nerve fiber in the plexiform dermal layer had a lower density (Fig. 1B). In the sixth post-operative month, free nerve endings in the epidermal layer (Fig. 1C) and nerve fibers with a higher density in the dermal layer were observed on the flap slice (Fig. 1D). A tactile corpuscle was observed in the dermal papillary layer (Fig. 2A). A Merkel cell was observed in the basal epidermal layer (Fig. 2B). The morphological integrity of the Pacinian corpuscle was observed in the deep dermal layer (Fig. 2C and D).

#### Statistical significance

The two-point discrimination data in the third and sixth post-operative months were 16.13±2.57 and 9.67±2.35 mm, respectively. A significant difference was observed between the two groups (P<0.05; Table II). The two-point discrimination recovery in the third post-operative month was improved compared with that in the sixth month.

## Discussion

Various senses of the skin, attributed to the sensory nerve endings and receptors, receive *in vivo* and *in vitro* stimulation, which are converted into certain action potentials along the nerve fiber. Different regions of the body have different skin sensations since the receptors in these parts and the density of the nerve fibers are different (2). The receptors contain free nerve endings and Merkel cells, as well tactile, Pacinian, Ruffini and Krause corpuscles. The free nerve endings detect pain sensation. Tactile corpuscles are for sensing tactile sensations, Pacinian corpuscles for pressure sensations and Krause corpuscles for temperature sensations. The static two-point discrimination test determined the density and function of the Merkel cell-axon complex. The regeneration of receptors is required for sensory recovery.

Previous studies evaluated the functional sensory recovery through bud anastomosis by chemotaxis. The bud regenerated and grew into a degenerative endoneurial tube and established contact with the sensory end organs (3–5). Manek *et al*(6) suggested that the nerve from the surrounding skin also grew along the degenerative endoneurial tube.

In this study, the flap slice in the third post-operative month showed minimal free nerve endings in the epidermal layer (Fig. 1A) and low density nerve fibers in the dermal layer (Fig. 1B), which indicate pain sensation recovery. The tactile sensation was also recovered; however, no tactile corpuscle was observed in the dermal papillary layer. Several studies (7) indicated that normal skin without tactile corpuscles may still experience tactile sensation, during which the free nerve endings primarily induce the sensation as opposed to the tactile corpuscle. The flap slice in the sixth post-operative month presented free nerve endings in the epidermal layer (Fig. 1C), as well as numerous nerve fibers with higher density in the dermal layer (Fig. 1D). This indicates an improved pain sensation recovery. Tactile corpuscles were observed in the dermal papillary layer (Fig. 2A). Morphological integrity of Pacinian corpuscles was observed in the deep dermal layer (Fig. 2C and D). A Merkel cell was observed in the basal epidermal layer (Fig. 2B). These results indicate that a significant recovery was achieved for tactile and pressure sensations, as well as the two-point discrimination of the flap, which corresponds to clinical function determination. No significant Krause corpuscles were observed in the slice due to the staining methods. This study failed to morphologically demonstrate the temperature sensation recovery.

The central route involves the regeneration of the nerve from the central flap area towards the edge (8). Clinical studies identified that the nerve-anastomosed flap demonstrated stronger capacities in scope and sensory recovery time since the growth of the proximal nerve along the nerve endoneurial tube induced skin flap sensory recovery. This phenomenon is known as the nerve contact guidance theory (9). Chang *et al*(10) used six different methods to reconstruct skin flap sensory function and the results of their study supported the above argument. The nerve-anastomosed flap was the flap with nerve implantation and sensory recovery through the central route. Daniel *et al*(11) found that the Tinel’s sign in the flap of nerve implantation exceeded the nerve anastomosis in the second post-operative month, which indicates nerve regeneration. The majority of flap areas demonstrated sensory recovery in the fifth post-operative month, which indicates that nerve anastomosis restored nerve continuity and created the conditions for flap sensory recovery. Gao (12) indicated that in nerve anastomosis of the flap, the regenerative nerve grows along the nerve distribution of the flap and arranges the nerve endings or sensory corpuscles. Li *et al*(13) also demonstrated that the flap with neural implantation obtains nerve reinnervation through the central route. However, studies (14) have shown that nerve anastomosis is only beneficial for the recovery of two-point discrimination and not for other sensory restoration. These studies hypothesized that neural anastomosis was only beneficial for Merkel cell-axonal complexes.

The peripheral route shows the opposite effect to the central route. In the peripheral route, the nerve regenerates at the base and edge of the flap and is concentrated in the central region. The traditional free flap, which is the flap without nerve anastomosis, receives reinnervation through the peripheral route.

Turko *et al*(15) examined a group of free flaps with denervation by extracting flap slices and observing their regeneration under an electron microscope. Sprouting axons regenerated from the periphery and base of the flap and grew into the flap through chemotaxis. Waris *et al*(16) conducted a study based on cholinesterase determination in the skin graft, whereby the skin graft obtained sensory recovery by peripheral nerve regeneration. By immunohistochemical analysis, Manek *et al*(6) observed in their animal experimental study that graft basal nerve regeneration succeeded peripheral tissue regeneration and that sensory fiber regeneration occurred first. Liu *et al*(17) concluded that the sensory nerve endings regenerate and the direction of nerve regeneration is from the peripheral to the central area.

In this present study, up to 30 flaps survived, with four of them positive for tactile sensation in the proximal area of the flap in the second post-operative week, while two flaps exhibited a partially restored pain sensation. This phenomenon was caused by the higher nerve density in the normal tissues near the flap that grew into the proximal area of the flap within a short period of time and formed free nerve endings in the epidermal layers of the flap, which are responsible for pain sensation. Another reason may be that in the early period of nerve regeneration, free nerve endings or residual tactile corpuscles without complete degeneration were responsible for tactile sensation instead of the tactile corpuscle. Denervation of the tactile corpuscle was shown to induce short-term nerve-ending degeneration without significantly changing the overall morphology (18), during which the residual tactile corpuscle played a certain role. In the first post-operative month, the majority of the flaps restored tactile sensation; however, a number of those in the central area failed to restore tactile sensation. More than half of the flaps restored pain sensation. The peripheral area of the 4 flaps restored cold and hot temperature sensation, whereas that of 3 flaps restored only cold sensation. Each flap with hot sensation recovery also restored cold sensation. These observations suggest that the cold sensation recovery of the flap was faster than the hot sensation.

In the third post-operative month, the central area of all flaps restored tactile and pain sensation, a number of which exhibited hyperalgesia. Only 2 flaps failed to restore temperature sensation. The two-point discrimination of 30 flaps demonstrated varying degrees of recovery. The majority of the flaps in the proximal and peripheral areas were more sensitive compared with those in the central area. In the sixth post-operative month, pain, tactile and temperature sensation, as well as the two-point discrimination of all flaps, were restored with a flap sensation function score of S3+, during which no statistically significant difference was indicated in sensation between the proximal or peripheral area and the center of the flap.

In this study, the phase recovery of tactile and pain sensations was faster in the second post-operative week up to the first post-operative month, whereas that of cold and hot sensations, as well as the two-point discrimination, was faster in the first up to the third post-operative month. The sensory recovery time of the flap was closely associated with the thickness of the flap (16,19,20). In our study, the sensory recovery time of the flap with thinning surgery was longer than that without surgery. Therefore, the abdominal free flap restored the sensation through the nerve in the peripheral and basal tissues, which grew onto the flap, known as the peripheral route. The direction of nerve regeneration was from proximal to distal, as well as from peripheral to central areas. The various sensory recovery sequences were as follows: the first recovery was tactile and pain sensation; the second recovery was cold sensation; the third recovery was hot sensation and the final recovery was two-point discrimination.

## Figures and Tables

**Table I.** Sensory function recovery of 30 flaps.

	Recovery of sensory function				
Time	Tactile	Pain	Cold	Hot	Two-point discrimination
2 weeks	4	2	0	0	0
1 month	25	18	7	4	0
3 months	30	30	28	23	30
6 months	30	30	30	30	30

**Table II.** Two-point discrimination recovery of 30 flaps.

Time (months)	Two-point discrimination (mm)
3	16.13±2.57
6	9.67±2.35

t=11.0221, P<0.05. Data are shown as the mean ± standard deviation.
